# Real-Time Visual Tracking through Fusion Features

**DOI:** 10.3390/s16070949

**Published:** 2016-06-23

**Authors:** Yang Ruan, Zhenzhong Wei

**Affiliations:** Key Laboratory of Precision Opto-Mechatronics Technology of Ministry of Education, Beihang University, Beijing 100191, China; ruanyang1987@163.com

**Keywords:** visual tracking, fusion feature, correlation filters

## Abstract

Due to their high-speed, correlation filters for object tracking have begun to receive increasing attention. Traditional object trackers based on correlation filters typically use a single type of feature. In this paper, we attempt to integrate multiple feature types to improve the performance, and we propose a new DD-HOG fusion feature that consists of discriminative descriptors (DDs) and histograms of oriented gradients (HOG). However, fusion features as multi-vector descriptors cannot be directly used in prior correlation filters. To overcome this difficulty, we propose a multi-vector correlation filter (MVCF) that can directly convolve with a multi-vector descriptor to obtain a single-channel response that indicates the location of an object. Experiments on the CVPR2013 tracking benchmark with the evaluation of state-of-the-art trackers show the effectiveness and speed of the proposed method. Moreover, we show that our MVCF tracker, which uses the DD-HOG descriptor, outperforms the structure-preserving object tracker (SPOT) in multi-object tracking because of its high-speed and ability to address heavy occlusion.

## 1. Introduction

Object tracking is an important computer vision task that has many practical applications, such as security and surveillance, motion analysis, augmented reality, traffic control and human–computer interaction. A real-time visual tracking system combines software and hardware design. A digital camera captures video. To achieve a smooth output video impression for human eyes, a frame-rate of at least 15 frames per second (FPS) is required. A two-axis turntable will be used to pivot the camera horizontally (yaw) and vertically (pitch). Object tracking will be accomplished through software on the main control computer. An interface allows the user to select a target and to see what the camera is tracking. The system attempts to always keep the object in the center of its field of view. It is noteworthy that target tracking algorithms play a decisive role in this system.

Single object tracking is the most common task within the field of computer vision. Many methods for object tracking have been proposed. Adam et al. [1] presented a part-based algorithm called FragTrack which models the object appearance based on multiple parts of the target. Grabner et al. [2] proposed an on-line boosting algorithm (OAB) to select features for tracking. In [3], Babenko et al. adopted Multiple Instance Learning (MIL), which puts all ambiguous positive and negative samples into bags to learn a discriminative model. Kalal et al. [4] proposed a novel tracking framework (TLD) that decomposes the tasks into three components: tracking, learning and detection. Struck [5] presents a framework for adaptive visual object tracking based on structured output prediction. Xu et al. [6] proposed the structural local sparse appearance (ASLA) model which exploits both partial information and spatial information. In [7], a robust tracking framework based on the locality sensitive histograms is proposed. Wang et al. [8] present a novel probability continuous outlier model (PCOM) to depict the continuous outliers that occur in the linear representation model. The approach [9] formulates the spatio-temporal relationships between the object of interest and its local context based on a Bayesian framework. In [10], Oron presented Extended Lucas Kanade or ELK that it casts the original LK algorithm as a maximum likelihood optimization. These methods rely on intensity or texture information for the image description and include complex appearance models and optimization methods. It is difficult for most of them, when executed on a standard PC, to keep up with the 25 frame-per-second demand without parallel computing when real-time processing is required [11].

Recently, correlation filters for object tracking began to receive more attention because they have an impressively high-speed. Several state-of-the-art methods using correlation filters have been proposed for a variety of applications, such as object detection and recognition and object tracking. Bolme et al. [12] propose a tracker that is based on the Minimum Output Sum of Squared Error (MOSSE) filter, which is robust to variations in lighting, scale, pose, and non-rigid deformations while operating at 669 frames per second. Henriques et al. [13] provide a link to Fourier analysis using the well-established theory of circulant matrices and devise Kernel classifiers with the same characteristics as the correlation. Their tracker is called a CSK tracker. Boddeti et al. [14] propose a vector correlation filter (VCF) with HOG features and demonstrate the efficacy and speed of the proposed approach on the challenging task of multi-view car alignment. Galoogahi et al. [15] propose an extension to canonical correlation filter theory that can efficiently handle multi-channel signals. In contrast to object tracking, color descriptors have been shown to obtain excellent results for object recognition and detection [16,17,18,19,20]. Most early color detectors use simple color representations for image description. The linguistic study of Berlin and Kay [21] on basic color terms is one of the most influential works in color naming. In [17], the authors show that the color names (CN) learned from real-world images outperform chip-based color names on real-world applications. Danelljan et al. [22] extend the CSK tracker [13] with color names (CN), which provides superior performance for visual tracking. Recently, Henriques et al. [23] derived a new Kernelized Correlation Filter (KCF), which is the journal version of CSK and can use HOG features very well.

**Figure 1:**
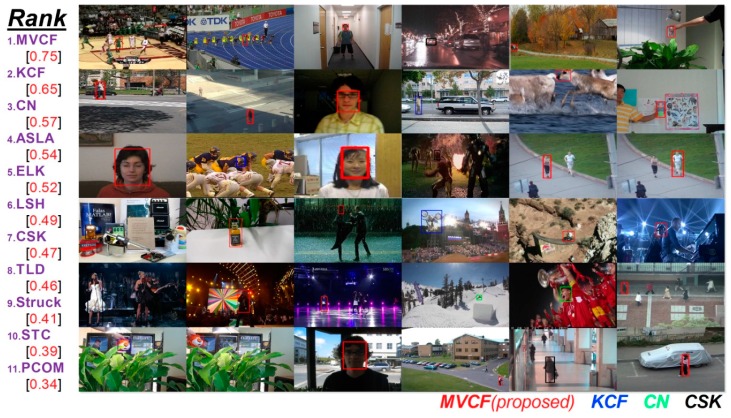
Tracking results in the CVPR2013 benchmark. We employ all 36 color sequences for evaluation. Note that there are two targets for the jogging sequence. Only the top tracker is presented in the corresponding color rectangle. The proposed tracker achieves the best performance in 26 of the 36 sequences. MVCF with the DD-HOG feature shows a significant improvement over state-of-the-art approaches using correlation filters, such as CSK with raw pixels, CN with color names and KCF with HOG. The quantitative comparison of our tracker with 10 state-of-the-art methods is reported in terms of its precision at a threshold of 20 pixels. The experimental results show that our approach outperforms state-of-the-art tracking methods.

Traditional object trackers based on correlation filters typically use a single type of feature. In this paper, we attempt to integrate multiple feature types to improve the performance. The fusion of multiple features leads to a significant increase in the performance for object detection [16,18]. In reference [16], the authors extend the description of the local features with color information. A boosted CN-HOG detector is proposed by [18], where CN descriptors are combined with HOGs to incorporate texture information. These investigators show that their approach can significantly improve the detection performance on the challenging PASCAL VOC datasets. Shi et al. [24] specifically show that the correct features to use are exactly those that make the tracker work best. To obtain an effective and efficient tracking algorithm, we propose a new DD-HOG fusion feature that consists of a discriminative descriptor (DD) and histograms of oriented gradients (HOG). Khan et al. [20] show that their discriminative descriptor (DD) outperforms other pure color descriptors and the color name (CN) descriptor. The DD feature has been used for object tracking [25]. In addition, Dalal and Triggs [26] proposed histograms of oriented gradients (HOG), which are widely used for object detection. However, the DD-HOG is a multi-vector descriptor and therefore cannot be directly used in prior correlation filters [12,13,14,15,16]. Those correlation filters have been traditionally designed to be used with scalar or single vector feature representations only. In our paper, we propose a multi-vector correlation filter (MVCF) to resolve this problem. A multi-vector correlation filter interpreted literally is made up of multiple vector correlation filters. The vector correlation filter is composed of one correlation filter. The DD-HOG feature is correlated with our multi-vector correlation filter for obtaining a single-channel response. The peak of the responses indicates the target center. A similar process can be done for other multi-vector features. The tracker that is based on a multi-vector correlation filter (MVCF) that comprises four main components: (1) a scale adjustment that makes all of the elements of a multi-vector feature have the same size; (2) a multi-vector structure that a multi-vector descriptor can be convolved with directly; (3) an update scheme that multiple object appearance models must update separately (and all of the previous frames are considered); and (4) a dimensionality reduction technique that reduces the dimension for each element of the multi-vector feature independently. A quantitative evaluation is conducted on the CVPR2013 tracking benchmark [11]. It is a comprehensive dataset that is specially designed to facilitate the evaluation of performance. Extensive experiments demonstrate that the proposed tracker based on the multi-vector correlation filter (MVCF) can outperform state-of-the-art trackers. Tracking results in the CVPR2013 benchmark are shown in Figure 1.

We also show that our MVCF tracker can obtain substantial performance in multi-object tracking. The goal of multi-object tracking is to estimate the states of multiple objects. In complex scenes, multi-object tracking remains a challenging problem for many reasons, including frequent occlusion by other objects, similar appearances of different objects, and real-time processing. In this paper, we argue that our MVCF tracker has an extraordinary ability to address partial occlusion and can run at an impressively high-speed. Therefore, the MVCF tracker appears to be a good choice for multi-object tracking. Our experimental evaluations show that the MVCF tracker performs very well on videos that are used [27] for multiple-object tracking. We use a simple approach to tracking multiple objects in which we only run multiple instances of our MVCF tracker without spatial constraints between the objects. The speed of our algorithm to track approximately four objects simultaneously is more than 25 fps. Therefore, the multi-vector correlation filter (MVCF) can be used as a basic framework in multi-object tracking such as Random Forests (RFs) and Support Vector Machines (SVMs).

The contributions of this paper are as follows.

**MVCF:** We propose a new type of correlation filter, a multi-vector correlation filter (MVCF), which can directly convolve with a multi-vector descriptor. Extensive experiments demonstrate that the proposed tracker, which is based upon MVCF, can outperform state-of-the-art trackers.

**Feature Selection:** We select optimal features to a multi-vector correlation filter based on how the tracker that uses MVCF works. The new proposed DD(11)-HOG fusion feature is the optimal feature for MVCF tracking. We also show that MVCF with DD(11)-HOG obtains superior performance compared with current state-of-the-art correlation filters with other features.

**Multi-object Tracking:** We apply our approach across multi-object tracking tasks. We demonstrate that MVCF is well suited to use as a basic framework in multi-object tracking, such as with RFs and SVMs. The speed of our algorithm for tracking approximately four objects simultaneously is more than 25 fps.

The remainder of this paper is organized as follows. In Section 2, we review the CSK tracker. In Section 3, we introduce a new robust tracker that is based on a multi-vector correlation filter. In Section 4, we show our experimental results. Finally, we present our conclusions in Section 5.

## 2. The CSK Tracker

Correlation filters have shown superior performance on a number of computer vision problems. The CSK tracker [13] is based on a kernelized single-channel correlation filter and runs at hundreds of frames per second. The key for its outstanding speed is that the CSK tracker exploits the circulant structure. The CSK tracker use scalar features, such as raw pixel values, and a grayscale image patch is preprocessed. The intensity channel is computed using Matlab’s “rgb2gray” function when the input is a 3-channel RGB color image. In this section, we describe briefly the CSK tracker.

### 2.1. Training Samples and Labels

Training Samples and Labels are used as inputs of the classifier. A classifier is trained using a single grayscale image patch x of size M×N that is centered around the target. The x is expressed as a MN×1 vector. The CSK tracker considers all of the cyclic shifts $x_{i}=P^{i}x$, i∈{(0,0)⋯,(M−1,N−1)}, which are referred to as training samples. The P is the permutation matrix that cyclically shifts vectors by one element to the right (the last element wraps around). The single grayscale image patch $x_{i}$ of size M×N is a training sample, and its corresponding confidence score is $y_{i}$. The label in a large majority of trackers is a binary value in general. In the CSK tracker, the labels that are computed by a Gaussian function are continuous values. The confidence score will be 1 nearby the target location ${\text{i}}^{\prime }=\left({\text{m}}^{\prime },{\text{n}}^{\prime }\right)$ and will decay to 0 as the distance increases. The total of all of the locations is M×N, which corresponds to the training sample. The label of the i=(m,n)th location is $y_{i}=\text{exp}\left(-\frac{0.5}{s^{2}}\times \left({\left(m-{\text{m}}^{\prime }\right)}^{2}+{\left(n-{\text{n}}^{\prime }\right)}^{2}\right)\right)$, where (m,n)∈{0,⋯,M−1}×{0,⋯,N−1} and the spatial bandwidth $s=\sqrt{\text{MN}}/16$.

### 2.2. Training

A classifier is trained by finding the parameter w that minimizes the cost function. The cost function minimization problem is written as

(1) $$\underset{w}{\min}\overset{M\times N}{\underset{j=1}{\sum }}{\left\|<\phi \left(x_{j}\right),w>-y_{j}\right\|}^{2}+{\lambda \|\text{w}\|}^{2}$$

where λ is a parameter for regularization, and the classifier has M×N pairs of training samples and labels.

The kernel is defined as $\kappa \left(x,{\text{x}}^{\prime }\right)=<\phi \left(x\right),\;\phi \left({\text{x}}^{\prime }\right)>$, where ϕ is the mapping to the Hilbert space. The inputs $x_{i}$ are mapped to a rich high-dimensional feature space using $\phi \left(x_{i}\right)$. The Representer Theorem [28] then states that the cost function in Equation (1) is minimized by the solution $w=\sum_{i}\alpha_{i}\phi \left(x_{i}\right)$, which can be expanded as a linear combination of the training samples. The parameter w and $\phi \left(x_{i}\right)$ have the same dimensionality. The online classifier coefficients α are updated by updating the solution w over time, where the coefficients α are

(2) $$ℱ\left(\alpha \right)=\frac{ℱ\left(y\right)}{ℱ\left(k_{x}\right)+\lambda }$$

where ℱ denote the Fourier transform, and the vector $k_{x}$ has elements $k_{i}=\kappa \left(x,P^{i}x\right)$, i=0,⋯,n−1. P is the permutation matrix.

In summary, the solution w is implicitly represented by the vector α, which is solved by Equation (2).

### 2.3. Fast Detection

In the new frame, a set of grayscale patches z of size M×N are obtained in a search region around an object location. The Kernel classifier can perform detection quickly with the Fast Fourier Transform (FFT). A classifier response is computed for each single input. All of the responses are evaluated simultaneously. The confidence map of a target center is obtained by

(3) $$\hat{\text{y}}={ℱ}^{-1}\left(ℱ\left(k_{z}\right)⊙ℱ\left(\alpha \right)\right)$$

where $k_{z}$ is a vector that has the elements ${\hat{\text{k}}}_{i}=\kappa \left(z_{i},\hat{\text{x}}\right)$, and ℱ and ${ℱ}^{-1}$ denote the Fourier transform and Fourier inverse transform, respectively, and ⊙ is the element-wise product. The learned object appearance $\hat{\text{x}}$ is updated overtime. The current model is computed by considering all of the previous frames. The best object location can be estimated by maximizing the confidence map.

## 3. Proposed Algorithm

The novelty of this paper is to present a real-time tracker that is based on the CSK algorithm. Our multi-vector correlation filter (MVCF) can directly use multi-vector descriptors (i.e., DD-HOG, CN-HOG). We present details of the proposed tracking algorithm in this section.

### 3.1. Input of Multi-Vector Correlation Filter

Generally, a unique vector is computed to represent an image patch when only one feature is used in a correlation filter framework. For a multi-vector descriptor, an image patch is mapped to multiple image representations. All of the descriptors carry with them corresponding mappings.

Given an image x, each vector feature is defined as $X^{i}=\varphi^{i}\left(x\right)$, i=1,2,⋯,ν, where ν is the size of the set of multi-vector features. Then, $X^{i}$ is the i-th vector feature, and its corresponding mapping is $\varphi^{i}$.

The multi-channel $X=\left[X^{1},{\text{ X}}^{2},\cdots ,X^{\nu }\right]$ is the input of the multi-vector correlation filter. The element $X^{i}=\left[\varphi^{i,1}\left(x\right),\varphi^{i,2}\left(x\right),\cdots ,\varphi^{i,D_{i}}\left(x\right)\right]$ is a $M\times N\times D_{i}$ tensor, where the $\varphi^{i,j}\left(x\right)$ is an M×N matrix in the $1\leq j\leq D_{i}$th channel of the ith element $X^{i}$. A fixed number for the channels is allotted for each element. The dimension D of the input X is $\sum_{i=1}^{\nu }D_{i}$, where the number of elements is ν. The multi-vector feature X as a whole is correlated with our multi-vector correlation filter.

It is necessary to consider the size of the different elements $X^{i}$. We try to use a pre-processing to ensure that all of the elements $X^{i}$ could have the same size M×N. In general, different features correspond with different elements $X^{i}$ by $\varphi^{i}=\left[\varphi^{i,1},\varphi^{i,2},\cdots ,\varphi^{i,D_{i}}\right]$. So, the dimensions of these elements are not the same. But this will not influence the pre-processing. Here, we give a briefing for the CN-HOG feature. Generally, an image is represented by HOG features which are computed densely. The cell is a 8 × 8 non-intersecting pixel region to represent an image. Of course, there are other n×n pixel cells used in practice. For the cell $c_{i}$, the representation is obtainedby concatenation, specifically, $c_{i}=\left[{\text{CN}}_{i},{\text{HOG}}_{i}\right]$. For the HOG descriptor, we first compute the intensity channel by the “rgb2gray” function, and then, the representation is computed in each cell (n×n pixel). The element $X^{\text{HOG}}$ has a decreasing size,  (M/n)×(N/n), and the dimension is 31. A similar procedure is built to compute the element $X^{\text{CN}}$ for each cell, resulting in the same size as the elements $X^{\text{HOG}}$. The RGB values are mapped to an 11-dimensional color representation. The bi-vector feature $X^{\text{CN}-\text{HOG}}$ as a whole has the size (M/n)×(N/n), and the dimension of the fusion vector is 42.

### 3.2. Multi-Vector Structure

**Figure 2:**
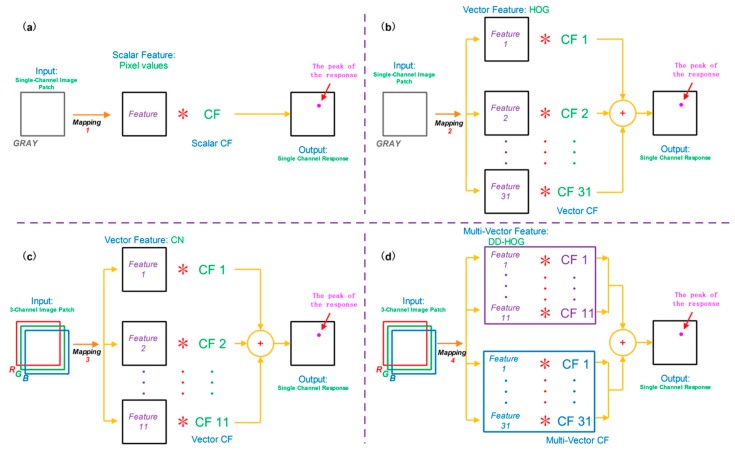
Different correlation filters were designed to be used with: pixel values (**a**); HOG (**b**); CN (**c**); and DD-HOG (**d**). To directly use multi-vector features, such as DD-HOG, we design a novel multi-vector structure.

To directly use multi-vector features, such as DD-HOG, we design a novel multi-vector structure in Figure 2. A multi-vector correlation filter, when interpreted literally, is composed of multiple vector correlation filters. The vector correlation filter is composed of a single correlation filter. For each feature channel, its corresponding confidence score is computed, and, an input patch $x_{j}$ are mapped using $\varphi^{i,d}\left(x_{j}\right)$ where i={1,2,⋯,ν} and $1\leq d\leq D_{i}$. Each input patch is collected around the target. The result $\varphi^{i,d}\left(x_{j}\right)$ is an M×N matrix in the $\text{d}+\sum_{n=1}^{i-1}D_{n}$, -th channel of the whole filter.

Equation (1) can then be expressed as

(4) $$\underset{w^{1,1},w^{1,2},\cdots ,w^{\nu ,D_{\nu }}}{\min}\underset{j}{\sum }{\left\|\overset{\nu }{\underset{i=1}{\sum }}\overset{D_{i}}{\underset{d=1}{\sum }}<\varphi^{i,d}\left(x_{j}\right),w^{i,d}>-y_{j}\right\|}^{2}+\lambda \overset{\nu }{\underset{i=1}{\sum }}\overset{D_{i}}{\underset{d=1}{\sum }}{\left\|w^{i,d}\right\|}^{2}$$

where the number of vectors is ν, and the number of channels of the ith vector is $D_{i}$. For each feature channel, there is a corresponding classifier. The confidence score indicates a similarity with the target. A sharp peak can be obtained near the target location. The final confidence score is attained by the aggregate of the outputs of each feature channel.

The aggregate is embodied in the kernel computation $\kappa \left(x,{\text{x}}^{\prime }\right)$. The manipulation of the linear kernel and Gaussian kernel are identical. The inputs x and ${\text{x}}^{\prime }$ as a whole vector feature are a M×N×D tensor, where M×N is the total number of locations, and D is the number of channels. The single channel k is obtained by the aggregate of the outputs of each feature channel. The notation k is a matrix of size M×N, and its dimension is one. Therefore, both the solution α in training and the confidence score vector $\hat{\text{y}}$ denote a M×N matrix.

### 3.3. Updating Scheme

The scheme needs to update both the learned object appearance $\hat{\text{x}}$ and the filter coefficients α overtime.

To update the model, all of the previous frames should be considered from the first frame until the current frame p. All of the previous appearances of the target are $\left\{x_{m}|m=1,\cdots ,p\right\}$. A positive weight constant $\beta_{m}$ is allocated for each frame. Here η is a learning rate parameter that can set the weight $\beta_{m}$. Equation (4) can then be expressed anew as

(5) $$\underset{w^{1,1},w^{1,2},\cdots ,w^{\nu ,D_{\nu }}}{\min}\overset{p}{\underset{m=1}{\sum }}\beta_{m}(\underset{j}{\sum }{\left\|\overset{\nu }{\underset{i=1}{\sum }}\overset{D_{i}}{\underset{d=1}{\sum }}<\varphi^{i,d}\left(x_{j,m}\right),w_{m}^{i,d}>-y_{j,m}\right\|}^{2}+\lambda \overset{\nu }{\underset{i=1}{\sum }}\overset{D_{i}}{\underset{d=1}{\sum }}{\left\|w_{m}^{i,d}\right\|}^{2})$$

where the number of vectors is ν, and the number of channels of the ith vector is $D_{i}$. λ is a parameter for regularization. In frame m, the corresponding confidence score of input image patch $x_{j,m}$ is $y_{j,m}$.

Then, the solution α in Equation (2) can then be expressed as

(6) $$ℱ\left(\alpha \right)=\frac{\sum_{m=1}^{p}\beta_{m}ℱ\left(y_{m}\right)ℱ\left(k_{m}\right)}{\sum_{m=1}^{p}\beta_{m}ℱ\left(k_{m}\right)\left(ℱ\left(k_{m}\right)+\lambda \right)}$$

This cost function is minimized by ℱ(α). The derivation of Equation (6) are given in Appendix.

The object appearance ${\hat{\text{x}}}^{P}=\left[{\hat{\text{x}}}_{1}^{p},{\hat{\text{x}}}_{2}^{p},\cdots ,{\hat{\text{x}}}_{\nu }^{p}\right]$ is an M×N×D tensor, where the number of vectors is ν, and ${\hat{\text{x}}}_{i}^{p}$ is an $M\times N\times D_{i}$ tensor. In each new frame, the filter is updated by

(7) $$\{\begin{array}{c} ℱ\left(\alpha \right)=U^{p}/V^{p}\\ U^{p}=\eta \overset{p}{\underset{m=1}{\sum }}\beta_{m}ℱ\left(y_{m}\right)ℱ\left(k_{m}\right)+\left(1-\eta \right)U^{p-1}\\ V^{p}=\eta \overset{p}{\underset{m=1}{\sum }}\beta_{m}ℱ\left(k_{m}\right)\left(ℱ\left(k_{m}\right)+\lambda \right)+\left(1-\eta \right)V^{p-1} \end{array}$$

The object appearance is updated by

(8) $${\hat{\text{x}}}_{i}^{p}=\left(1-\eta \right){\hat{\text{x}}}_{i}^{p-1}+{\eta \text{x}}_{i}^{p},\;\text{i}=1,2,\cdots ,\nu$$

### 3.4. Dimension Reduction

For the current frame p, the object appearance ${\hat{\text{x}}}^{P}=\left[{\hat{\text{x}}}_{1}^{p},{\hat{\text{x}}}_{2}^{p},\cdots ,{\hat{\text{x}}}_{\nu }^{p}\right]$ consists of ν object sub-appearances. For each sub-appearance ${\hat{\text{x}}}_{i}^{p}$, i=1, 2, ⋯,ν, we use an eigenvalue decomposition technique (EVD) independently, which reduces the dimension to obtain a boosted speed.

For the first frame, we extract the image patch using the initial ground truth. The multi-vector $X_{p}=\left[X_{p}^{1},{\text{X}}_{p}^{2},\cdots ,{\text{X}}_{p}^{\nu }\right]$ is the input of the multi-vector correlation filter mentioned in Section 3.1, where p is the current frame. For each multi-channel vector feature $X_{1}^{i}$, its corresponding covariance matrix $A_{i}^{1}$ is computed, where i=1, 2,⋯,ν. Covariance matrix $A_{i}^{1}$ is a square matrix of size $D_{i}\times D_{i}$. Then, we perform an eigenvalue decomposition of the matrix $A_{i}^{1}$. The covariance matrix is decomposed to the following form: $A_{i}^{1}=Q_{i}^{1}\Sigma_{i}^{1}{\left(Q_{i}^{1}\right)}^{T}$, where $Q_{i}^{1}$ is composed of eigenvectors of the covariance matrix $A_{i}^{1}$, and the sorted eigenvalues are stored in a diagonal matrix $\Sigma_{i}^{1}$. Let ${{\text{D}}^{\prime }}_{i}$ be a planned low dimension, and ${{\text{D}}^{\prime }}_{i}\in \{1,\cdots ,D_{i}|i=1,\cdots ,\nu \}$. Then, $E_{i}^{1}$ is a ${{\text{D}}^{\prime }}_{i}\times {{\text{D}}^{\prime }}_{i}$ diagonal matrix of the eigenvalues from $\Sigma_{i}^{1}$. The projection matrix $B_{i}^{1}$ is selected as the first ${{\text{D}}^{\prime }}_{i}$ in $Q_{i}^{1}$. The low-dimensional sub-appearance ${\hat{\text{x}}}_{i}^{1}$ is obtained by ${\hat{\text{x}}}_{i}^{1}=X_{1}^{i}B_{i}^{1}$, where i=1, 2, ⋯,ν, and the dimension of ${\hat{\text{x}}}_{i}^{1}$ is ${{\text{D}}^{\prime }}_{i}$. The learned appearance ${\hat{\text{x}}}^{1}=\left[{\hat{\text{x}}}_{1}^{1},{\hat{\text{x}}}_{2}^{1},\cdots ,{\hat{\text{x}}}_{\nu }^{1}\right]$ is used to compute the detectionscores $\hat{\text{y}}$ for the next frame. In each new frame, the covariance matrix that is ready for EVD is updated by ${\hat{\text{A}}}_{i}^{p}=\left(1-\mu \right)C_{i}^{p-1}+{\mu \text{A}}_{i}^{p}$, where $C_{i}^{p}=\left(1-\mu \right)C_{i}^{p-1}+{\mu \text{B}}_{i}^{p}E_{i}^{p}{\left(B_{i}^{p}\right)}^{T}$ and $C_{i}^{1}=B_{i}^{1}E_{i}^{1}{\left(B_{i}^{1}\right)}^{T}$. The procedure is similar for the subsequent frames.

### 3.5. Main Differences from CSK, CN and KCF

All types of correlation filters are designed depending on the usage of a feature. The searching for and usage of good features are a significant part of the methodology.

Traditionally, many different correlation filters were designed to be used with scalar feature (most commonly pixel value) representations only. The CSK tracker uses this traditional type of correlation filter, which is a single channel correlation filter. The CN tracker proposes a tracking method that can handle multi-channel color feature vectors (color name descriptors). The vector correlation filter (VCF) used in the CN tracker was designed by Boddeti [14] and it is a multi-channel correlation filter. The journal version of CSK (called KCF) also uses the VCF and has already been able to address the HOG features very well. The KCF selects the HOG features to obtain better performance.

Reference [18] shows that the simple fusion of CN and HOG obtains an outstanding performance increase for object detection. We propose a new type of fusion feature (DD-HOG) that can gain a significant improvement in performance for tracking. The multi-vector descriptor cannot be directly used in prior correlation filters. We design a multi-vector correlation filter (MVCF) to solve this difficulty. The MVCF can also use the other multi-vector descriptors readily. Table 1 shows detailed information about the differences between our method and the above three methods.

Here, we briefly analyze the main factor for why the proposed approach is better than previous ones. In [29], they find that the feature extractor plays the most important role in a tracker. On the other hand, the observation model often brings no significant improvement. Thus, selecting the right features provides the potential for improving performance. Using the proposed sophisticated fusion features can dramatically improve the tracking performance. This feature could make the correlation filters tracking system work better.

## 4. Experiments

In this section, we present qualitative and quantitative tracking results. We performed two sets of experiments to evaluate the performance of our tracker. In the first set of experiments, we selected an optimal fusion feature for our multi-vector correlation filter, used a dimensionality reduction technique for the optimal feature and compared our tracker with other existing state-of-the-art trackers. Moreover, our tracker is compared to other correlation-based methods, such as CSK [13], CN [22] and KCF [23]. In the second set of experiments, we evaluated the performance of the MVCF tracker for multi-object tracking.

### 4.1. Evaluation Setup

The proposed tracker is implemented in Matlab on a workstation with a 3.7 GHz processor and 8 GB RAM without sophisticated program optimization. In our approach, the parameters are fixed for all of the sequences. A Gaussian kernel is used in our tracker. We refer to the parameters of the proposed model in [13,22,23]. We set the bandwidth of the Gaussian kernel to σ=0.5, the spatial bandwidth to $s=\sqrt{\text{MN}}/16$ for a target of size M×N, regularization to $\lambda =10^{-4}$, adaptation parameter to μ=0.15, and learning rate to η=0.2. For all of the fusion features that use HOG, we use 4 × 4 pixel cells.

We use two criteria, the tracking precision and success rate, as quantitative evaluations [11].

**Precision:** The center location error (CLE) is a tracking evaluation method that is widely used; it is defined as the distance between the central locations of the tracked target and the manually labeled ground truths. The precision score shows the percentage of frames whose estimated location is within the given threshold distance of the ground truth. The default threshold is equal to 20 pixels.

**Success Rate:** Another evaluation method is the Pascal VOC overlap ratio (VOR). The overlap score is defined as $S=\frac{\left|r_{t}\cap^{\text{​}}r_{a}\right|}{\left|r_{t}\cup^{\text{​}}r_{a}\right|}$, where $r_{t}$ represents the tracked bounding box, and $r_{a}$ represents the ground truth bounding box. The ∩ and ∪ represent the intersection and union of two regions, respectively, and |⋅| denotes the number of pixels in the region. If this overlap rate is above a given threshold, then the tracking result in one frame is considered to be a success. The default threshold is equal to 50%. The success rate is computed with all of the frames.

### 4.2. Color Descriptors

We describe the color descriptors that we will use to augment the HOG feature descriptors for object tracking. In the following description, we refer to some of the published articles [16,18,19,20,22,26,30]. Table 2 shows a comparison of the feature dimensionality of the different color descriptors.

#### 4.2.1. Color Representations

**RGB:** The standard 3-channel RGB color space, which is by far the most commonly used color space.

**HSV:** H is for the hue, S is for the saturation, and V is for the value. It is often more natural to think about a color in terms of its hue and saturation than in terms of the additive or subtractive color components.

**HSL:** The HSL (hue, saturation, lightness/luminance) is quite similar to HSV, with “lightness” replacing “brightness”. It is also known as HSI (hue, saturation, intensity).

**YCbCr:** YCbCr is approximately perceptually uniform and is used as a part of the color image pipeline in video and digital photography systems.

**LAB:** In the Lab color space, the dimension L is for the lightness, and A and B are for the color-opponent dimensions.

**Opponent:** This representation is invariant with respect to specularities, and the image is transformed by $\left(\begin{array}{l} O1\\ O2\\ O3 \end{array}\right)=\left(\begin{array}{ccc} \frac{1}{\sqrt{2}} & \frac{-1}{\sqrt{2}} & 0\\ \frac{1}{\sqrt{6}} & \frac{1}{\sqrt{6}} & \frac{-2}{\sqrt{6}}\\ \frac{1}{\sqrt{3}} & \frac{1}{\sqrt{3}} & \frac{1}{\sqrt{3}} \end{array}\right)\;\left(\begin{array}{c} R\\ G\\ B \end{array}\right)$.

**C:** The C color representation adds photometric invariants with respect to shadow-shading to the opponent descriptor by normalizing by the intensity. This step is performed according to C=(O1O3 O2O3 O3)T.

**CN:** Color names, or color attributes, are linguistic color labels that humans assign to colors in the world. Berlin and Kay [21], in a linguistic study, concluded that the English language contains eleven basic color terms: black, blue, brown, gray, green, orange, pink, purple, red, white and yellow. In this paper, we use the mapping in [17], which is automatically learned from Google images.

#### 4.2.2. Bi-Vector Descriptors

**CN-HOG:** Among various features, the histograms of oriented gradients (HOG) proposed by Dalal and Triggs [26] are the most commonly used features for object detection. A single-channel grayscale image/signal is mapped to a 31 dimensional image/signal representation. Generally, an image is represented by HOG features that are computed densely. The cell is an 8 × 8 non-intersecting pixel region to represent an image. Of course, there are other n × n pixel cells used in practice. A 31-dimensionalvector is computed to represent each cell. Although both memory usage and time complexity rise, the discriminative ability of the HOG-based classifier increases compared with the classifier using raw pixel values. The authors of [18] incorporate 11-dimensional color names into a 31-dimensional HOG feature, which results in increased performance. Due to the cell computing of HOG, there is a similar procedure to compute the color attributes for each cell.

**DD(11)-HOG, DD(25)-HOG:** We extend the 31-dimensional HOG vector with the discriminative descriptor (DD) vector that is proposed by Khan et al. [20] to obtain an outstanding discriminative power in a classification problem. The discriminative descriptor (DD) is not limited to eleven color names and can freely choose the desired dimensionality. The authors of [20] make the universal color descriptors available for settings with 11, 25, and 50 clusters. Our goal is to obtain a compact and discriminative descriptor, and thus, we consider only DD(11) and DD(25). DD(25) outperforms all of the other descriptors (CN, DD(11) and DD(50)) that were used in their experiment. We hope to know whether similar results can be obtained for object tracking.

#### 4.2.3. Tri-Vector Descriptors

**RGB-HOG, HSV-HOG, HSL-HOG, YCbCr-HOG, LAB-HOG, OPP-HOG, and C-HOG:** The seven color spaces mentioned are from 3-channel color space. The HOG feature is a 31-dimensional image representation. The fusion extensions of HOG based on computing the HOG on multiple color channels result in a dimensionality of 93. An example is furnished to explain the fusion. For RGB, the representation is RGB=[R, G, B]. For RGB-HOG, the representation is obtained by concatenation: RGB-HOG=[R-HOG, G-HOG, B-HOG], and the dimensionality of this concatenated representation is 93.

### 4.3. Evaluation on Comprehensive Benchmark

The first set of experiments is conducted on the CVPR2013 tracking benchmark [11], which is specially designed for the evaluation of the tracking performance. The tracking dataset consists of 50 fully annotated sequences, which provide a large number of scene changes and target motions. There are many challenging factors in these sequences, including illumination change, scale change, occlusion, and fast motion.

#### 4.3.1. Experiment 1: Feature Selection

We first perform an experiment to select the optimal features for the multi-vector correlation filter. The performance of the color descriptors is evaluated on the task of object tracking. Table 3 shows the results on all 36color videos of the CVPR2013 tracking benchmark. The results are reported in terms of both the precision, with a CLE of 20 pixels, and success rate, with a VOR of 50%. We also provide the median frames per second (FPS). For HOG features with 4 × 4 pixel cells, the intensity channel is computed using Matlab’s “rgb2gray” function.

Table 3 also shows a comparison of the feature dimensions of different color descriptors. The features on the top are more compact than the features on the bottom. The fusion features CN-HOG and DD(11)-HOG have a dimensionality 42. This dimensionality is significantly more compact than a fusion approach in which the HOG would be computed on multiple color channels. The results clearly show that the DD(11)-HOG descriptor performs best with a 0.68 success rate and 0.75 precision. In [20], the DD descriptor with 25 dimensions outperforms all of the other descriptors, including the 11-dimensional descriptor and CN descriptor for object detection. However, the DD(11)-HOG obtains the best results in our experiment. Moreover, the fusion approach in which the HOG would be computed on multiple color channels cannot obtain good results. CN-HOG provides the highest speed among the ten color descriptors. The median speed of DD(11)-HOG over all 36 sequences is 72 fps. In general, MVCF provides a high speed regardless of what features are used. In summary, DD(11)-HOG is the optimal feature for the multi-vector correlation filter for tracking.

#### 4.3.2. Experiment 2: Low-Dimensional DD(11)-HOG Feature

In simple fusion, we incorporate 11-dimensional color names (DD) into a 31-dimensional HOG feature, such as CN-HOG in [18]. The dimension of the fusion feature DD(11)-HOG is 42. The computational time scales linearly with the dimension of the fusion feature. In this paper, we use an adaptive dimensionality reduction technique that reduces the dimension of the multi-vector separately. The technique is applied to compress the 42-dimensional DD(11)-HOG to only nine dimensions of DD(11)_5_-HOG_4_, including five-dimensional DD(11) and 4-dimensional HOG. Table 4 shows the results that were obtained using the DD(11)-HOG and its comparison with other features. The CSK tracker uses a single-channel correlation filter with raw pixels, the CN tracker uses a vector correlation filter with the CN descriptor, and the KCF tracker uses the vector correlation filter with the HOG descriptor. The quantitative evaluation shows that the MVCF with DD(11)-HOG obtains the best results, successfully tracks objects in almost all of the sequences in this dataset, and outperforms the other three methods, including CSK, CN and KCF. The results also show that our MVCF with the DD(11)_5_-HOG_4_ feature further improves the speed without a significant loss in the accuracy. If we use a greater compression ratio in the dimensionality reduction technique, both the success rate and precision score gradually decrease. Among the five trackers, CSK is shown to provide the highest speed. The KCF obtains the second fastest speed. The speed of MVCF using DD(11)-HOG is faster than the CN tracker, which also uses a color descriptor. The proposed tracker based on MVCF can outperform prior state-of-the-art trackers using correlation filters.

#### 4.3.3. Experiment 3: Comparison with State-of-the-Art Tracking

We compare our algorithm with state-of-the-art online tracking methods, including the TLD [4], Struck [5], ASLA [6], LSH [7], PCOM [8], ELK [10], STC [9], CSK [13], CN [22] and KCF [23]. For a fair comparison, we use the source code that is provided by the authors with tuned parameters to obtain the best performance.

**Figure 3:**
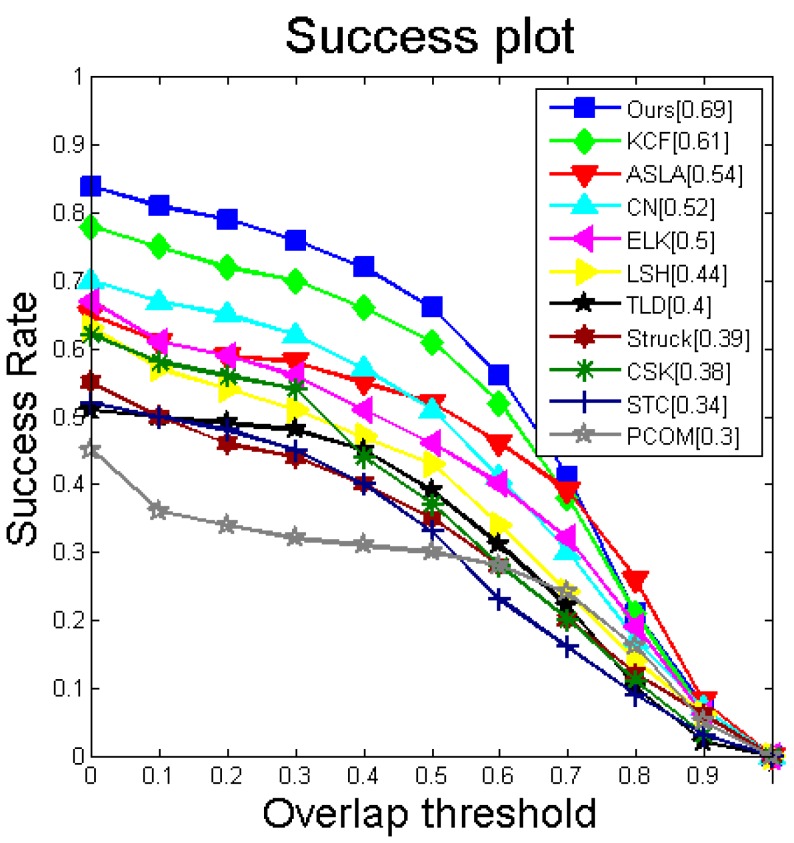
Success plot over all 36 sequences. The scores of each tracker are reported in the legends. The trackers are ranked from top to bottom. The values in the legend are the mean VOR at 0.5.

Our tracker produces an overall performance that is comparable to state-of-the-art trackers, as presented in Table 5. Our method is ranked in first place for its overall performance on this benchmark dataset, with a success rate of 0.69 and a precision score of 0.75. Our tracker has a high speed, which is 90 fps. A tracker that needs to address live video streams must have a high processing speed. In general, a frame-rate of at least 25 fps is required. However, the speed of many algorithms in our experiment is fewer than 20 fps. Figure 3 shows the success plot over all of the 36 sequences. The results are reported at a success rate with a VOR of 50%. Our algorithm achieves the best results.

For better evaluation and analysis of the strengths and weaknesses of the tracking approaches, the authors of [11] annotate the sequences with 11 different attributes, namely, illumination variation (IV), scale variation (SV), occlusion (OCC), deformation (DEF), motion blur (MB), fast motion (FM), in-plane rotation (IPR), out-of-plane rotation (OPR), out-of-view (OV), background clutter (BC) and low resolution (LR). By annotating the attributes of each sequence, they construct subsets that have different dominant attributes, which facilitates analyzing the performances of the trackers for each challenging factor.

**Figure 4:**
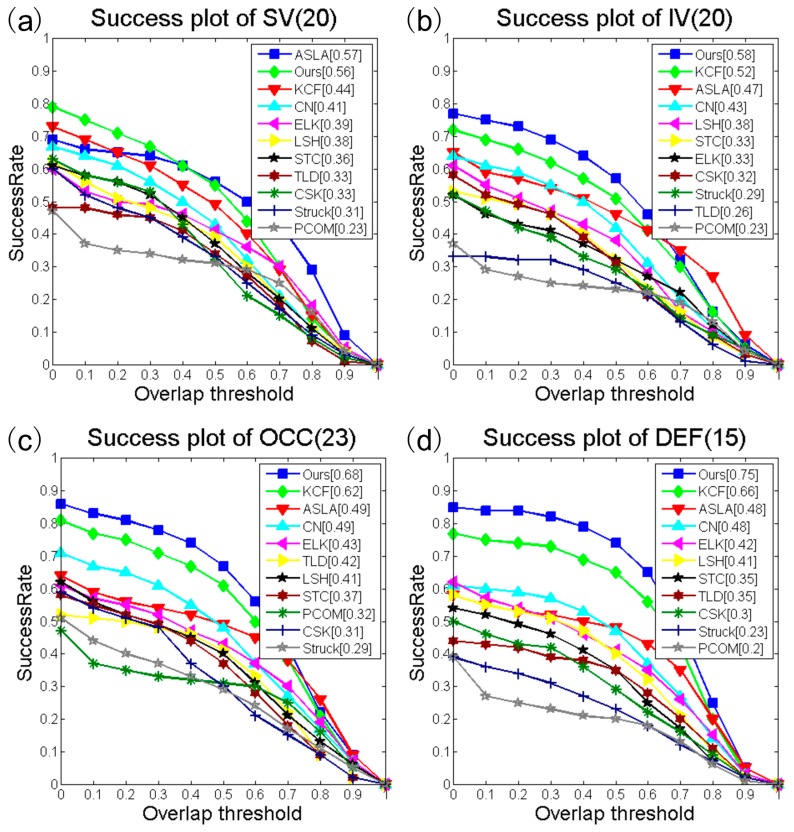
Success rate plots for IV (**a**); SV (**b**); OCC (**c**) and DEF (**d**) subsets. The value appears in the title is the number of sequences in that subset.

**Figure 5:**
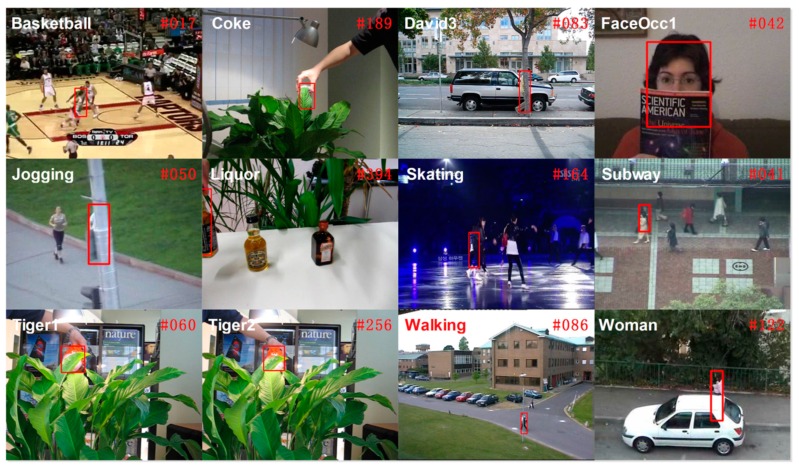
Tracking results of our tracker in the heavily occluded object tracking dataset. In most of these sequences, the occluded part of the target is above 50%. The experimental results show that our tracker can handle occlusion well.

Table 6 presents the success rate and our tracker rank for the different sequence attributes in the benchmark dataset. Some of the trackers perform well on a few subsets. However, our method outperforms the others on most of the subsets. Our tracker achieves the best performance in 10 of the 11 subsets. On the SV subset, the ASLA method performs better than ours. The ASLA approach with scale adaptation is the best, achieving a success rate of 0.57 while the success rate of our tracker is 0.56. Even with a fixed scale, our tracker is robust to appearance variations that are introduced by scale variations. Owing to space restrictions, we only illustrate the success plots for attributes illumination variation (IV), scale variation (SV), occlusion (OCC) and deformation (DEF) as shown in Figure 4. Both Table 6 and Figure 5 demonstrate that the proposed tracker has an extraordinary ability to address heavy occlusion.

### 4.4. Multiple-Object Tracking

We first evaluate the performance of our tracker on the videos used in [27] for multi-object tracking. These nine videos include multiple objects, and the average length of the videos is 842 frames. We compare the performance of our MVCF tracker with the mst-SPOT tracker. The basis of the structure-preserving object tracker (SPOT) is formed by the popular Dalal-Triggs detector [26], which was obtained by training a linear SVM on HOG features. In their experiments, the mst-SPOT tracker outperforms the other baseline trackers (OAB, TLD, and no-SPOT) in almost all of the videos. We use a simple approach for tracking multiple objects that only runs multiple instances of our MVCF tracker without spatial constraints between the objects.

**Figure 6:**
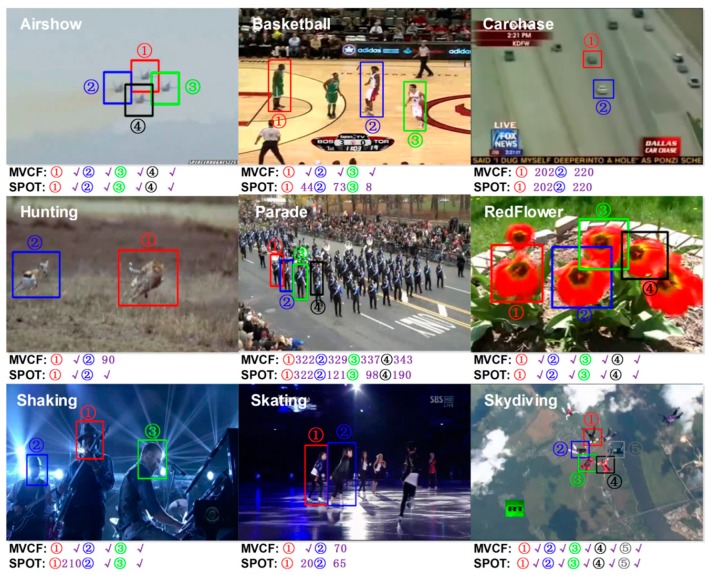
Performance of our MVCF tracker and the SPOT tracker on Multiple-Object Videos. There are 29 single object tasks in all nine videos. Trackers that can successfully tracks objects in all of the frames of the sequence are denoted by a “√”. If a tracker misses the object, then we provide the frame number, which denotes the last frame that can be tracked successfully. MVCF outperforms SPOT, and MVCF can successfully track objects in almost all of the sequences without spatial constraints between the objects. In the Carchase and Parade sequences, MVCF can track the targets until they leave the view. The speed of our algorithm to track approximately four objects simultaneously is more than 25 fps.

We evaluate the performance of the trackers by measuring the precision and success rate of each tracker and averaging over five runs. Table 7 and Figure 6 depict the tracking results of the SPOT tracker and ours, evaluated on nine videos. In this experiment, the proposed MVCF achieves overall the best performance using both the precision and success rate. The computational complexity grows linearly in the number of objects being tracked. The speed of our algorithm to track approximately four objects simultaneously is more than 25 fps.

Moreover, we qualitatively describe the results. There are 29 single object tasks in all nine videos. Our tracker successfully tracks objects in almost all of the sequences in this dataset. Only two objects, gazelles in the Hunting video and Dancer 2 in the skating video, cannot be tracked well by our tracker. The gazelle is very fast in Hunting and undergoes significant pose changes. It is unlikely that online appearance models are able to adapt fast and correctly. In the Carchase and Parade sequences, MVCF can track the targets until they leave the view. The SPOT tracker fails to track some of the objects, including three players in the basketball video, three persons in the parade video, Singer 1 in the shaking video and two dancers in the skating video. This finding clearly shows that our approach delivers competitive results, even though it does not consider the spatial constraints between objects.

## 5. Conclusions

In this paper, we propose a robust tracker that is based on a multi-vector correlation filter (MVCF) that can efficiently handle multi-vector fusion features. We propose a new DD(11)-HOG fusion feature using our MVCF tracker, which leads to a significant increase in the performance for object tracking. Numerous experimental results and evaluations demonstrate that the proposed tracker can outperform existing state-of-the-art trackers in the literature. Moreover, we argue that our MVCF tracker has a powerful ability to address partial occlusion and can run at an impressively high-speed. Therefore, MVCF is an ideal framework for multi-object tracking. We hope this work can motivate other researchers to perform more in-depth study in other computer vision tasks.

## Figures and Tables

**Table 1:** Comparison of different approaches.

Method	Descriptors	Correlation Filter	Number of Channels	Video Type
CSK [13]	Pixel values	Scalar	Single	Grey scale
CN [22]	Color Name(CN)	Single-Vector	Multiple	Color
KCF [23]	HOG	Single-Vector	Multiple	Grey scale
Ours	DD(11)-HOG	Multi-Vector	Multiple	Color

**Table 2:** Comparison of feature dimensionality of different color descriptors.

Feature	Dimension
CN-HOG	42
DD(11)-HOG	42
DD(25)-HOG	56
RGB-HOG, HSV-HOG, HSL-HOG,YCbCr-HOG,LAB-HOG, OPP-HOG, C-HOG	93

**Table 3:** The achieved performance evaluated by the Precision/Success Rate/FPS.

Method	Dimensions	Success Rate	Precision	FPS
CN-HOG	42	0.66	0.73	80.9
DD(11)-HOG	42	0.68	0.75	72.0
DD(25)-HOG	56	0.66	0.72	50.7
RGB-HOG	93	0.43	0.49	75.7
HSV-HOG	93	0.46	0.54	56.3
HSL-HOG	93	0.46	0.52	51.0
YCbCr-HOG	93	0.40	0.49	59.1
LAB-HOG	93	0.44	0.52	43.0
OPP-HOG	93	0.36	0.46	65.3
C-HOG	93	0.47	0.55	49.6

**Table 4:** The results of different trackers using correlation filters.

Method	CSK [13]	CN [22]	KCF [23]	DD(11)-HOG	DD(11)_5_-HOG_4_
SuccessRate	0.38	0.52	0.61	0.68	0.69
Precision	0.47	0.57	0.65	0.75	0.75
FPS	164	66	151	72	90

**Table 5:** Quantitative comparison of our tracker with 10 state-of-the-art methods over 36 challenging sequences. The Results are reported in terms of both the median success rate and precision. We also provide the median frames per second (FPS).

Method	Success Rate	Precision	FPS
PCOM [8]	0.3	0.34	18
STC [9]	0.34	0.39	98
Struck [5]	0.39	0.41	22
TLD [4]	0.4	0.46	21
LSH [7]	0.44	0.49	7
ELK [10]	0.5	0.52	6
ASLA [6]	0.54	0.54	7
CSK [13]	0.38	0.47	164
CN [22]	0.52	0.57	66
KCF [23]	0.61	0.65	151
Ours	0.69	0.75	90

**Table 6:** Our tracker success rate and rank for different sequence attributes.

Attribute	IV	SV	OCC	DEF	MB	FM	IPR	OPR	OV	BC	LR
Number of Sequences	20	20	24	16	10	13	20	28	4	18	4
Success Rate	0.58	0.56	0.68	0.75	0.66	0.55	0.66	0.63	0.47	0.68	0.4
Rank	1	2	1	1	1	1	1	1	1	1	1

**Table 7:** Qualitative results on Multiple-Object Videos for the proposed MVCF, compared with SPOT.

Sequence	MVCF			SPOT [27]		
	Success Rate	Precision	Median FPS	Success Rate	Precision	Median FPS
Air Show	0.88	0.97	34	0.83	0.97	7
Basketball	0.95	0.96	15	0.20	0.20	2
Car Chase	0.63	0.8	62	0.61	0.80	4
Hunting	0.53	0.48	20	0.91	0.61	5
Parade	0.86	0.97	45	0.34	0.50	6
Red Flowers	0.96	0.93	13	0.98	0.92	5
Shaking	0.94	0.92	21	0.61	0.57	3
Skating	0.69	0.65	15	0.39	0.32	3
Sky Diving	0.97	1.00	26	0.95	0.97	2
Average	0.82	0.85	27.9	0.64	0.65	4.1

## Appendix

In this section, we try to prove that the cost Function (5) is minimized by choosing the coefficients as Equation (6). In order to make the derivation more easy to understand, we use $\varphi =\left[\varphi^{1,1},\cdots ,\varphi^{1,D_{1}},\cdots ,\varphi^{v,1},\cdots ,\varphi^{v,D_{v}}\right]$ and $w=\left[w^{1,1},\cdots ,w^{1,D_{1}},\cdots ,w^{v,1},\cdots ,w^{v,D_{v}}\right]$ to simplify the cost Function (5) in the paper.

$$\epsilon =\overset{p}{\underset{m=1}{\sum }}\beta_{m}\left(\underset{j}{\sum }{\left\|<\varphi \left(x_{j,m}\right),w_{m}>-y_{j,m}\right\|}^{2}+\lambda {\left\|{\text{w}}_{m}\right\|}^{2}\right)$$

$$w_{m}=\underset{l}{\sum }\alpha_{l}\varphi \left(x_{l,m}\right)$$

This equation is rewritten to

$$\epsilon =\overset{p}{\underset{m=1}{\sum }}\beta_{m}\left(\underset{j}{\sum }{\left(\underset{l}{\sum }\alpha_{l}\kappa \left(x_{j,m},x_{l,m}\right)-y_{j,m}\right)}^{2}+\lambda \underset{j}{\sum }\alpha_{j}\underset{l}{\sum }\alpha_{l}\;\kappa \left(x_{j,m},x_{l,m}\right)\right)$$

The derivative with respect to $\alpha_{r}$ is computed and the * indicates circular convolution.

$$\begin{array}{l} \frac{\partial \epsilon }{\partial \alpha_{\text{r}}}=2\sum_{\text{m}=1}^{\text{p}}\beta_{\text{m}}\sum_{\text{j}}\kappa ({\text{x}}_{\text{j},\text{m}},{\text{x}}_{\text{l},\text{m}})(\sum_{\text{l}}\alpha_{\text{l}}\kappa ({\text{x}}_{\text{j},\text{m}},{\text{x}}_{\text{l},\text{m}})-{\text{y}}_{\text{j},\text{m}}+\lambda \alpha_{\text{j}}\\ \;=2\sum_{\text{m}=1}^{\text{p}}\beta_{\text{m}}\sum_{\text{j}}\kappa ({\text{x}}_{\text{r}-\text{j},\text{m}},{\text{x}}_{\text{m}})(\sum_{\text{l}}\alpha_{\text{l}}\kappa ({\text{x}}_{\text{l}-\text{j},\text{m}},{\text{x}}_{\text{l}})-{\text{y}}_{\text{j},\text{m}}+\lambda \alpha_{\text{j}}\\ \;=2\sum_{\text{m}=1}^{\text{p}}\beta_{\text{m}}\sum_{\text{j}}\kappa_{\text{r}-\text{j},\text{m}}(\sum_{\text{l}}\alpha_{\text{l}}\kappa_{\text{l}-\text{j},\text{m}}-{\text{y}}_{\text{j},\text{m}}+\lambda \alpha_{\text{j}})\\ \;=2\sum_{\text{m}=1}^{\text{p}}\beta_{\text{m}}\sum_{\text{j}}\kappa_{\text{r}-\text{j},\text{m}}(\alpha *\kappa_{\text{j}.\text{m}}-{\text{y}}_{\text{j},\text{m}}+\lambda \alpha_{\text{j}})\\ \;=2\sum_{\text{m}=1}^{\text{p}}\beta_{\text{m}}\kappa_{\text{m}}*(\alpha *\kappa_{\text{m}}-{\text{y}}_{\text{m}}+\lambda \alpha ) \end{array}$$

Then, we set this derivative to zero,

$$\begin{array}{l} \frac{\partial \epsilon }{\partial \alpha_{r}}=0\Leftrightarrow \sum_{\text{m}=1}^{\text{p}}\beta_{\text{m}}\kappa_{\text{m}}-y_{\text{m}}+\lambda \alpha )=o\\ \Leftrightarrow ℱ\left\{\sum_{\text{m}=1}^{\text{p}}\beta_{\text{m}}\kappa_{\text{m}}*(\alpha *\kappa_{\text{m}}-y_{\text{m}}+\lambda \alpha )\right\}=0\\ \Leftrightarrow \sum_{\text{m}=1}^{\text{p}}\beta_{\text{m}}ℱ(\kappa_{\text{m}})(ℱ(\alpha )ℱ(\kappa_{\text{m}})-ℱ({\text{y}}_{\text{m}})+\lambda ℱ(\alpha ))=0\\ \Leftrightarrow \sum_{\text{m}=1}^{\text{p}}\beta_{\text{m}}ℱ(\kappa_{\text{m}})\left(ℱ(\kappa_{\text{m}})+\lambda \right)-\sum_{\text{m}}^{\text{p}}\beta_{\text{m}}ℱ(y_{\text{m}})ℱ(\kappa_{\text{m}})=0\\ \Leftrightarrow ℱ(\alpha )=\frac{\sum_{\text{m}=1}^{\text{p}}\beta_{\text{m}}ℱ({\text{y}}_{\text{m}})ℱ(\kappa_{\text{m}})}{\sum_{\text{m}=1}^{\text{p}}\beta_{\text{m}}ℱ(\kappa_{\text{m}})(ℱ(\kappa_{\text{m}})+\lambda )} \end{array}$$
